# PoachNet: Predicting Poaching Using an Ontology-Based Knowledge Graph

**DOI:** 10.3390/s24248142

**Published:** 2024-12-20

**Authors:** Naeima Hamed, Omer Rana, Pablo Orozco-terWengel, Benoît Goossens, Charith Perera

**Affiliations:** 1 School of Computer Science and Informatics, Cardiff University, Cardiff CF24 4AG, UK; ranaof@cardiff.ac.uk (O.R.); pererac@cardiff.ac.uk (C.P.); 2 School of Biosciences, Cardiff University, Cardiff CF10 3AX, UK; orozco-terwengelpa@cardiff.ac.uk (P.O.-t.); goossensbr@cardiff.ac.uk (B.G.)

**Keywords:** wildlife, poaching, knowledge graph, deep learning, predictive analytics

## Abstract

Poaching poses a significant threat to wildlife and their habitats, necessitating advanced tools for its prediction and prevention. Existing tools for poaching prediction face challenges such as inconsistent poaching data, spatiotemporal complexity, and translating predictions into actionable insights for conservation efforts. This paper presents PoachNet, a novel predictive system that integrates deep learning with Semantic Web reasoning to infer poaching likelihood. Using elephant GPS data extracted from an ontology-based knowledge graph, PoachNet employs a sequential neural network to predict future movements, which are semantically modelled and incorporated into the graph. Semantic Web Rule Language (SWRL) is applied to infer poaching risk based on these geo-location predictions and poaching rule-based logic. By addressing spatiotemporal complexity and integrating predictions into an actionable semantic rule, PoachNet advances the field, with its geo-location prediction model outperforming state-of-the-art approaches.

## 1. Introduction

Habitat loss, human–elephant conflict, and poaching threaten Bornean elephants (*Elephas maximus*) [1]. Despite global anti-poaching efforts, the illegal ivory trade continues to drive poaching, reducing the population to fewer than 1500 [2,3,4]. In Sabah, Malaysian Borneo, over 200 elephants died between 2010 and 2021, many through poisoning near oil palm plantations [5,6]. High-profile incidents, such as the 2013 poisoning of 14 pygmy elephants, highlight the escalating conflict between expanding agriculture and wildlife conservation.

Other species, including Bornean orangutans (*Pongo pygmaeus*), proboscis monkeys (*Nasalis larvatus*), and Sunda pangolins (*Manis javanica*), face similar threats from habitat loss and animal trafficking for illegal trade [6]. Poaching also endangers human lives, with rangers facing armed poachers [7].

Scarce and inconsistent poaching data complicate reliable predictions [8], prompting researchers to leverage environmental data for insights. For example, GPS sensors used by the World Wildlife Fund in Sabah help monitor elephant behaviour and mitigate human-elephant conflicts. Advanced machine learning models built on GPS and environmental data can predict wildlife movements, assisting targeted anti-poaching efforts.

Studies like those by Chibeya et al. [9] and Fang et al. [10] have demonstrated the potential of combining sparse data with computational techniques to predict poaching hotspots. However, challenges remain in integrating diverse datasets with advanced algorithms to improve prediction accuracy.

This paper introduces PoachNet, a predictive tool designed to address these challenges by integrating wildlife data with advanced algorithms. PoachNet employs deep learning with an ontology-based knowledge graph, creating a dynamic and hybrid model for poaching prediction. Elephant GPS observations are processed through a sequential neural network to predict geo-locations, which are semantically modelled and incorporated into the knowledge graph. Semantic Web Rule Language (SWRL) asserts poaching rules based on events not explicitly expressed in the data (ontology-based knowledge graph). PoachNet’s performance was benchmarked against state-of-the-art methods and demonstrated higher accuracy, consistently outperforming them.

The remainder of this paper is structured as follows: Section 2 reviews some related work. Section 3 includes the methodology for developing the ontology-based knowledge graph. Section 4 introduces the elephant geo-location prediction model. Section 5 presents the results. Section 6 discusses the results. Section 7 concludes the paper.

## 2. Related Work

In this section we survey some existing literature about knowledge graphs in predictive modelling and crime prediction. We also discuss recent wildlife crime prediction methods.

### 2.1. Knowledge Graphs for Data Modelling

Knowledge graphs (KGs) have seen significant advancements, particularly in enhancing predictive models. Pahuja et al. [11] addressed challenges in traditional Graph Neural Networks (GNNs), such as over-smoothing and scalability, by proposing a “retrieve-and-read” framework using a Transformer-based GNN to improve contextual predictions. Similarly, Duan and Chiang [12] developed an ontology-driven system to streamline predictive tasks by integrating diverse data into KGs.

In urban planning, Ning [13] introduced the Unified Urban Knowledge Graph (UUKG), containing millions of data triplets for two cities, to enhance spatiotemporal predictions. The study revealed complex structural patterns in UrbanKGs, tested KG embedding methods in predictive models, and made the dataset publicly available. Yan [14] applied virtual KGs for predictive analytics in hydraulic systems, aligning with Industry 4.0 needs, particularly in predictive maintenance.

In healthcare, Feng et al. [15] introduced DKADE, combining deep learning and KGs to detect adverse drug events (ADEs) while addressing gaps in clinical narratives. Zeng et al. [15] reviewed KG applications in drug discovery and ADE prediction, while Wang [16] developed KG-DTI, a KG-driven deep learning model for drug-target interaction predictions in Alzheimer’s disease treatment. These methods demonstrate the transformative potential of KGs across fields like urban planning, industrial systems, and healthcare.

### 2.2. Knowledge Graphs for Crime Prediction

Tompson et al. [17] highlighted the value of integrating Open Data for crime prediction, emphasizing the role of knowledge graphs in organizing and enriching data with semantics for better decision-making, as explored by Sikos [18]. Deepak et al. [19] used a Bi-LSTM neural network to classify crimes based on Google News and Twitter data, integrating ontologies dynamically crafted from weighted graphs of news and social media sources.

Wang et al. [20] developed HAGEN, a graph convolutional recurrent network that predicts crime across regions by leveraging homophily-aware constraints for similar crime patterns. Iqbal et al. [21] employed Naïve Bayesian methods, Decision Trees, and Confusion Matrices to classify crimes using U.S. crime datasets, while Bogomolov et al. [22] combined mobile network, demographic, and crime data to forecast hotspots in London using Logistic Regression, Neural Networks, and Random Forests.

In other studies, Almanie et al. [23] analyzed crime datasets from Denver and Los Angeles with Decision Trees and Bayesian methods, and Chen et al. [24] used Twitter and weather data with linear models to predict crimes. Kang et al. [25] integrated crime, demographic, weather, and image data using a deep neural network (DNN) with feature-level data fusion, enhancing predictions with environmental context insights.

### 2.3. Wildlife Crime Prediction

Efforts in wildlife conservation have been bolstered by major data platforms like the Global Biodiversity Information Facility (GBIF) [26], the Encyclopedia of Life (EOL) [27], Wikidata [28], and eBird [29], which aggregate and provide accessible biodiversity records. These resources play a vital role in addressing wildlife crime, a transnational issue involving syndicates exploiting biodiversity for illegal gains.

Research and data science have addressed various aspects of wildlife crime, including wildfire prediction, by employing advanced techniques such as deep learning and data compression [30] to enhance prediction accuracy and reduce computational costs [31]. For poaching crimes, Hofer et al. [32] explored it from an economic point of view, while Bakana et al. [33] focused on multimedia data mining for poacher detection. Haas et al. [34] employed federated databases to disrupt wildlife trafficking networks and later developed a political-ecological model to guide conservation decisions in poaching-prone areas [35]. Critchlow et al. [36] used ranger patrol data with Bayesian models to map illegal activities, emphasizing the predictive value of past crime locations over ecological covariates.

Technological advancements have strengthened anti-poaching efforts. Gore et al. [37] emphasized geospatial data standards to enhance data sharing and wildlife crime analysis. Yang et al. [38] introduced PAWS, a game theory-based ranger patrol optimization tool, while Nguyen et al. [39] improved upon it with CAPTURE, which addressed observational and temporal uncertainties in poaching prediction. CAPTURE’s limitations, such as interpretability and learning efficiency, were addressed by Kar et al. [40] with INTERCEPT, which employed spatially aware decision trees and ensemble models to improve prediction accuracy.

**Figure 1:**
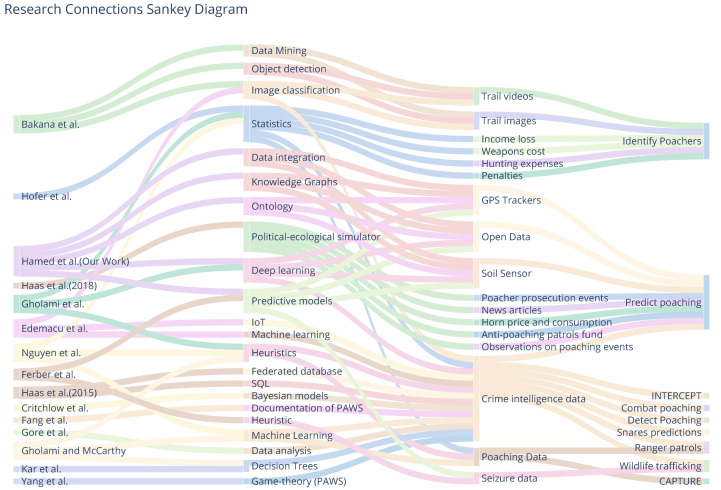
Multi-level Sankey Diagram to visualise Wildlife crime prediction authors, their approach, data used and the resultant product. Bakana et al. [33], Hofer et al. [32], Haas et al. [34], Edemacu et al. [42], Ferber et al. [43], Haas et al. [35], Gore et al. [37], Hamed et al. (PoachNet), Critchlow et al. [36], Gholami et al. [44], Kar et al. [40], Yang et al. [38], Fang et al. [45], Nguyen et al. [39], Gholami and McCarthy [41].

Gholami et al. [41] further advanced the field by combining CAPTURE and INTERCEPT into a hybrid spatiotemporal model, enabling precise hotspot prediction and more effective ranger deployment. Their iWare-E1 model, trained on 14 years of wildlife crime data, achieved remarkable success in detecting poaching activities in protected areas. Figure 1 illustrates various past research efforts addressing poaching and compares them with our approach.

Building on these foundations, this work introduces PoachNet, a novel approach that integrates deep learning and an ontology-based knowledge graph. By capturing complex animal behaviours and incorporating reasoning rules, PoachNet outperforms traditional and state-of-the-art models.

## 3. Methodology

This section discusses how PoachNet was created. The process begins with constructing a knowledge graph using the Forest Observatory Ontology (FOO) (w3id.org/def/foo#). Following this, spatiotemporal predictions were performed and subsequently incorporated into the ontology-based knowledge graph, enhancing its capacity for reasoning about poaching.

### 3.1. Ontology-Based Knowledge Graph

**Figure 2:**
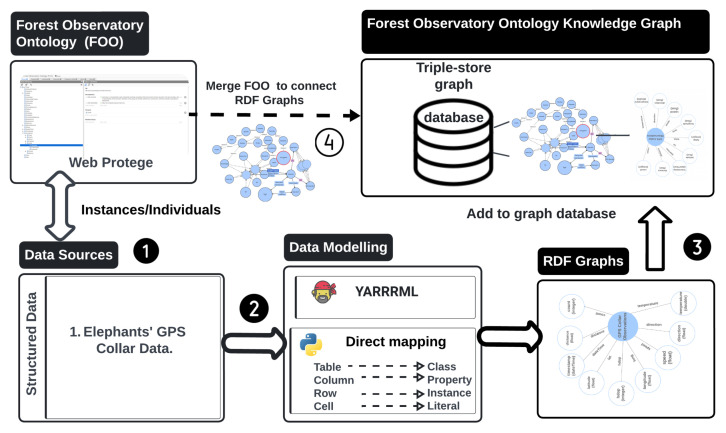
The proposed ontology-based knowledge graph construction approach.

**Figure 3:**
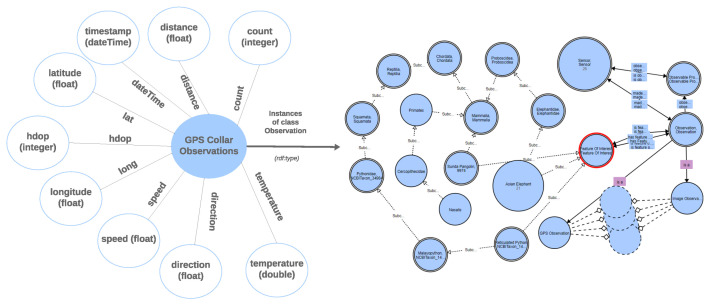
The ontology-based knowledge graph lightweight version or conceptual model. FOO was visualized using the WebVOWL tool (version 1.1.7) available at (https://service.tib.eu/webvowl/).

The Forest Observatory Ontology (FOO) integrates and links heterogeneous Resource Description Framework (RDF) graphs to create ontology-based knowledge graph(s). Figure 2 illustrates the generation process of an ontology-based knowledge graph for this study, while Figure 3 shows a lightweight representation of the resulting knowledge graph. FOO was developed using the Protégé platform and enriched with elephant Seri GPS observation variables. To construct the knowledge graph, we employed YARRML, a mapping tool described by Van Assche et al. [46], which uses FOO’s URI to connect external data entities as fragments (i.e., appending them to the URI with a hash (#)). The mapper processed GPS collar data from a CSV file to create an RDF knowledge graph using the Forest Observatory Ontology (FOO). It parses fields such as local and GMT dates, times, latitude, longitude, temperature, speed, and activity, mapping them to semantic entities like sensor observations and linking them to their sensor (GPS) and its features of interest (i.e., elephants).

### 3.2. Study Hub

Our study modelled data from the Lower Kinabatangan Wildlife Sanctuary in Sabah, Malaysian Bornean. This sanctuary, spanning about 270 km² and situated between E 118°00′–118°50′, N 5°20′–5°50′, features a tropical rain-forest climate and is home to a variety of endangered species (e.g., Bornean elephants (*Elephas maximus*), orangutans (*Pongo pygmaeus*), and Sunda pangolins (*Manis javanice*). The data obtained for this area comprised animal tracking sensor data.

### 3.3. Elephant Tracker Data

Elephant tracker data were sourced from Danau Girang Field Centre [47], which contained Global Positioning System (GPS) collars on adult Bornean elephants (*Elephas maximus*). The collars, provided by Africa Wildlife Tracking (awt.co.za), recorded data every two hours from 2012 to 2018, including location, time, temperature, and speed. These 14 kg collars, equipped with a GPS receiver and VHF transmitter, were fitted by researchers, trackers, and wildlife practitioners.

Table 1 describes 9168 observations from a GPS tracking collar attached to an elephant named Seri. Metrics include latitude (lat), longitude (long), temperature (Temperature), external temperature (ExtTemp), activity, speed, direction, covariance (Cov), horizontal dilution of precision (HDOP), distance, and count. The latitude and longitude data show minimal variation, with averages of 5.20° and 118.66°, respectively, indicating a specific geographic area. The mean temperature is 29.20 °C, with a wide range from −37.00 °C to 60.50 °C, suggesting potential anomalies or extreme environmental conditions. Metrics such as external temperature, activity, speed, and direction show uniformity, with all values at 0, possibly indicating static conditions or missing data. Covariance and HDOP have average values of 1.23 and 2.21, respectively, highlighting variable GPS signal quality. Distance values range widely, with an average of 273.89 units, and the Count metric spans from 2199 to 11,366, indicating diverse data recording frequencies. The historical elephant Seri data were first converted into RDF format and merged with FOO in a triplestore (Stardog) to form the ontology-based knowledge graph containing 202,885 triples.

## 4. PoachNet Geo-Location Prediction

We developed a neural network model to predict geo-location attributes based on data extracted from the ontology-based knowledge graph. The selected features—localDate, localTime, latitude, and longitude—were chosen for their critical role in capturing both temporal and spatial dimensions of movement. Temporal features such as localDate and localTime provide essential information about when an event or movement occurred, while latitude and longitude define the exact geographic location, making them key predictors for modelling movement patterns. These features were extracted from the knowledge graph using the rdflib library and SPARQL queries.

### 4.1. Data Extraction and Preprocessing

We employed SPARQL Protocol and RDF Query Language [48] (Listing 1) to retrieve relevant attributes from the RDF graph. The query extracted geo-location data points representing observations for latitude, longitude, date, and time. The extracted data were converted into numerical values for further preprocessing.

**Listing 1.** Query to extract elephant geo-location data including latitude, longitude, date, and time.


SELECT *

{?observation  a <https://w3id.org/def/foo# *gPSObservation* > *;*

    <http://www.w3.org/2003/01/geo/wgs84_pos# *latitude* > *?lat;*
    <http://www.w3.org/2003/01/geo/wgs84_pos#*longitude*> *?long;*
    <https://w3id.org/def/foo# *localDate* > *?localDate;*

    <https://w3id.org/def/foo# *localTime* > *?localTime. }*



The retrieved features were then processed to generate feature vectors (‘day’, ‘month’, ‘year’, ‘hour’) and label vectors (‘latitude’, ‘longitude’). The ‘NumPy’ library was used to convert these vectors into float-compatible arrays, enabling compatibility with the neural network. The dataset was split into training, validation, and testing subsets. Initially, 20% of the data was reserved for testing, while the remaining 80% was split further into 60% training and 20% validation sets.

### 4.2. Architecture and Training

A sequential neural network model was developed using TensorFlow and Keras to predict continuous target variables. The model architecture consisted of an input layer that accepted four features (‘date’, ‘time’, ‘longitude’, and ‘latitude’), followed by two hidden layers with 128 and 64 neurons, respectively. Each hidden layer used the Rectified Linear Unit (ReLU) activation function to learn non-linear patterns in the data. The output layer employed a linear activation function, enabling precise predictions of the target values (‘date’, ‘time’, ‘longitude’, and ‘latitude’).

**Figure 4:**
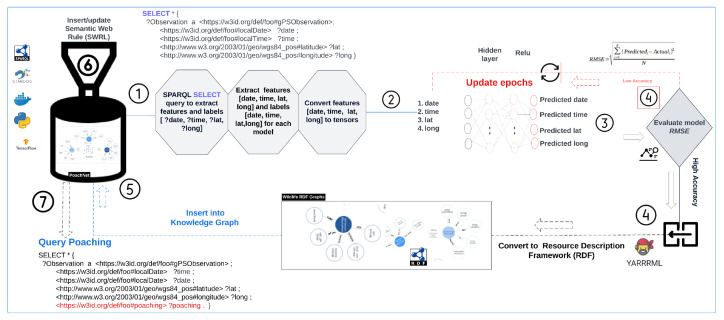
PoachNet: End-to-end predictive framework featuring RDF data extraction and deep learning and showcasing the integration of the ontology-based knowledge graphs with deep learning. The framework consists of a sequential neural network for predicting an elephants future geo-location. The network comprises an input layer with a shape matching the dataset’s four features, followed by two hidden layers employing the Rectified Linear Unit (ReLU) activation function. The output layer uses a linear activation function. The model’s performance was assessed using the Root Mean Square Error (RMSE) metric, and accurate predictions were mapped back to their original RDF format.

The model was compiled using the Adam optimizer and Root Mean Squared Error (RMSE) as the loss function, quantifying prediction accuracy. The model training was conducted on 500 epochs with a batch size of 32, incorporating validation data to monitor performance and mitigate overfitting. The trained model achieved precise geo-location predictions, evaluated using RMSE. The predictions were transformed into RDF graphs and integrated into the ontology-based knowledge graph, enriching it for rule-based reasoning. Figure 4 illustrates the predictive framework, and the pseudocode in Algorithm 1 outlines the entire workflow.
**Algorithm 1:** Data Extraction and Regression Model Training
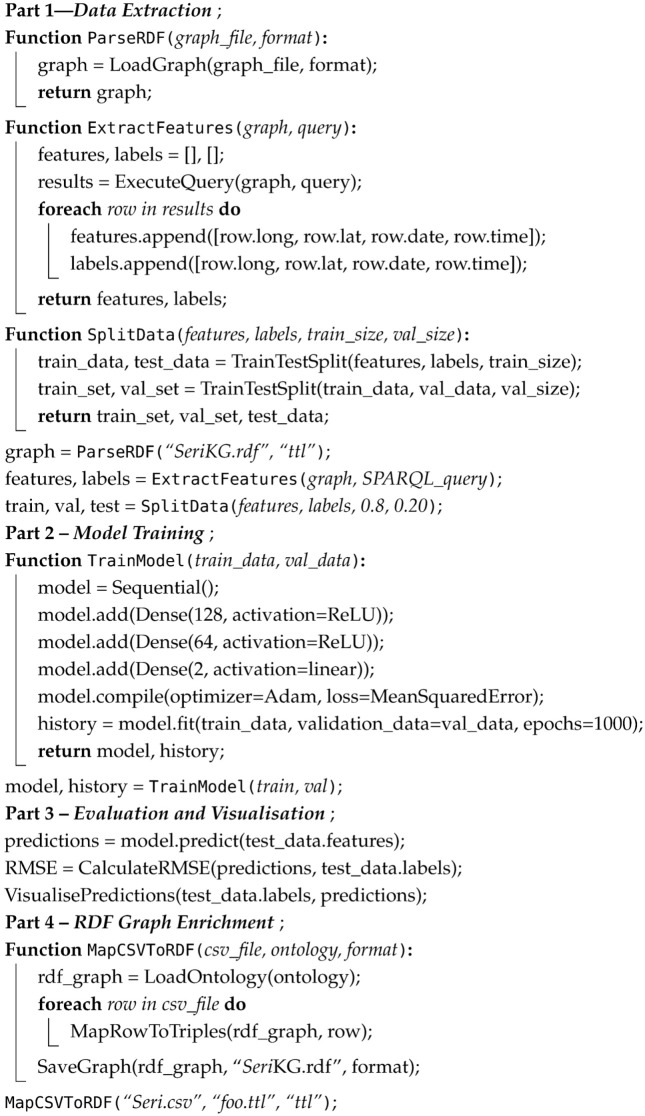


## 5. Reasoning for Poaching Prediction

Borenean elephants eat diverse vegetation, including palm leaves (https://borneomammals.online/2018/10/15/bornean-elephant-feeding-in-oil-palm-at-tabin; https://rainforest-rescue.org/petitions/905/malaysia-pygmy-elephants-poisoned-for-palm-oil accessed on 11 December 2024) [49,50]. Monitoring their diet and vegetation choices helps identify danger and poaching hotspots (e.g., oil palm plantations). To predict poaching, we used the Semantic Web Rule Language (SWRL). That is, creating a rule to insert in the ontology-based knowledge graph—inspired by insights from biologists and conservationists at Danau Girang Field Centre (DGFC).

Our experiment created a specific rule (refer to Rule Listing 2) to anticipate poaching activities. This rule predicts poaching based on an elephant’s proximity to a designated hazardous area, like an oil palm plantation. The criterion states that if an elephant equipped with a GPS tracker and termed elephant Seri is near oil palm plantations—areas marked as hazardous owing to previous poaching/poisoning incidents—then there is an increased likelihood that the elephant will be poached. Rule-based semantic reasoning ability led to a new binary poaching indicator in the knowledge graph database (triple store), where ‘1’ indicates potential poaching and ‘0’ indicates its absence.

**Figure 5:**
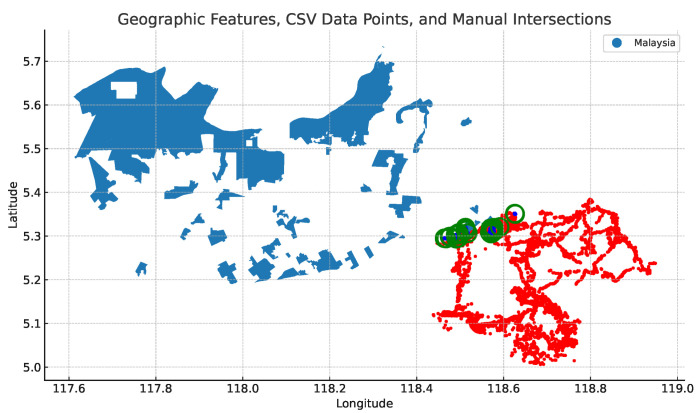
The map shows the geo-points (in green) intersection between the oil palm plantation (in blue) and the elephant movements (in red).

**Figure 6:**
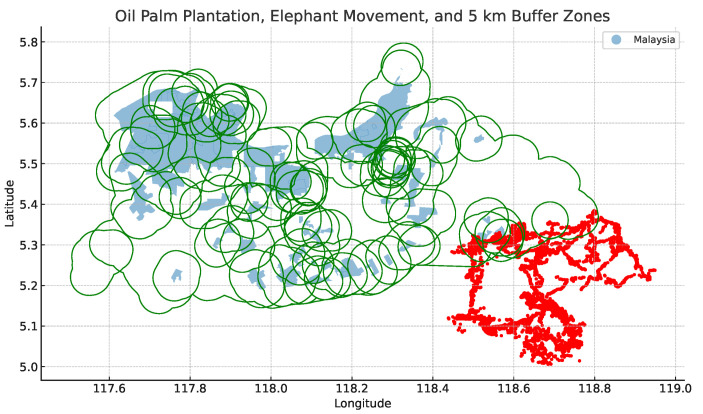
The figure displays a 5 km buffer zone (in green) around each geographic feature of an oil palm plantation. These zones are overlaid with the original geographic features (shown with slight transparency) and the points from the elephant movements (in red).

To determine if an elephant is within a 5 km radius of the oil palm location (see Figure 5), we created buffer zones with a radius of 5 km between the oil palm plantation and the elephant geo-location. Figure 6 visualises the 5 km buffer zones used to formulate the semantic rule.

The haversine formula was applied to calculate the distance between the two points. The elephant can be marked as potentially poached if the calculated distance is less than or equal to 5 km. The Haversine formula is given by this equation:

$$\begin{array}{c} a=\sin^{2}\left(\frac{\Delta \mathrm{lat}}{2}\right)+\cos\left({\mathrm{lat}}_{1}\right)\cdot \cos\left({\mathrm{lat}}_{2}\right)\cdot \sin^{2}\left(\frac{\Delta \mathrm{long}}{2}\right)\\ c=2\cdot \mathrm{atan}2\left(\sqrt{a},\sqrt{1-a}\right)\\ d=R\cdot c \end{array}$$

where Δlat is the difference in latitude, Δlong is the difference in longitude, ${\mathrm{lat}}_{1}$ and ${\mathrm{lat}}_{2}$ are the latitudes of the two points, *R* is the radius of the Earth (mean radius = 6371 km), and *d* is the distance between the two points.

**Listing 2.** Poaching Rule.


 


INSERT {

  ?s **a** <https://w3id.org/def/foo# *gPSObservation* > *;*

         <https://w3id.org/def/foo# *poaching* > *?poaching.*

}

**WHERE** {

?s **a** <https://w3id.org/def/foo# *gPSObservation* > *;*

       <http://www.w3.org/2003/01/geo/wgs84_pos# *latitude* > *?lat;*

       <http://www.w3.org/2003/01/geo/wgs84_pos# *longitude* > *?long.*


 


  # *Retrieve plantation details*

  <https://w3id.org/def/foo# *plantation* > *a* < *https://w3id.org/def/foo#OilPalmPlantation* > *;*

                                        <http://www.w3.org/2003/01/geo/wgs84_pos# *latitude* > *?plantationLat;*

                                        <http://www.w3.org/2003/01/geo/wgs84_pos# *longitude* > *?plantationLong.*


 


  # *Convert coordinates to float (if stored as literals)*

  BIND(xsd:float(?lat) AS ?latitude)

  BIND(xsd:float(?long) AS ?longitude)

  BIND(xsd:float(?plantationLat) AS ?oilpalmLat)

  BIND(xsd:float(?plantationLong) AS ?oilpalmLong)


 


  # *Calculate distance using the Haversine formula*

  BIND(6371 * 2 * ASIN(SQRT(

    POW(SIN((?latitude - ?oilpalmLat) * PI() / 180 / 2), 2) +

    COS(?oilpalmLat * PI() / 180) * COS(?latitude * PI() / 180) *

    POW(SIN((?longitude - ?oilpalmLong) * PI() / 180 / 2), 2)

  )) AS ?distance)


 


  # *Determine poaching based on the calculated distance*

  BIND(IF(?distance <= 5, 1, 0) AS ?poaching. }



## 6. Results

This section presents the results of PoachNet. The foundation of this approach is the ontology-based knowledge graph, now publicly accessible online. However, the elephant Seri GPS Observations dataset used in this research is kept confidential due to its sensitive nature. The graph injected with Semantic Web Rule Language (SWRL) enabled the semantic reasoning about poaching and introduced new triples to assert the poaching likelihood (Listing 3). The query results fed into the deep learning models demonstrated high accuracy and compatibility with machine learning formats. To evaluate the accuracy of the geo-location predictions, the Root Mean Square Error (RMSE) was used. The deep learning model was trained using Tensorflow in Google Colab. The computer hosting the model is a Dell Latitude 4520, equipped with an 11th Gen Intel(R) Core(TM) i7-1165G7 processor @ 2.8GHz (8 CPUs) and 16GB of DDR4 RAM, sourced from the United Kingdom.

**Listing 3.** Query to retrieve poaching status in the format of turtle graph with the geo-location coordinates, local data and poaching likelihood.


 

**CONSTRUCT WHERE** {
  ?Observation  a  <https://w3id.org/def/foo# *gPSObservation* > *;*
         <https://w3id.org/def/foo#*localDate*>   *?LocalDate* *;*         <http://www.w3.org/2003/01/geo/wgs84_pos#*latitude*> *?lat* *;*
         <http://www.w3.org/2003/01/geo/wgs84_pos# *longitude* > *?long ;*

         <https://w3id.org/def/foo# *poaching* > *?poaching.  }*


 




### 6.1. PoachNet Geo-Locations Prediction Result

The proposed neural network model for the geo-location prediction is a linear model and was built using the TensorFlow and Keras frameworks. The model contained an input layer intended to accommodate four critical features (data, time, longitude and latitude) related to the geographical positioning of elephant Seri. The network also includes two subsequent dense layers, containing 128 and 64 neurons, respectively, using the Rectified Linear Unit (ReLU) activation function to capture non-linear patterns in the data effectively. In other words, the model is an output layer with two neurons, employing a linear activation function. Such configuration is well-suited for regression tasks of continuous outputs (i.e., longitude and latitude). The data used contained 9168 observations, and their distribution is shown in Figure 4 step 3. This model underwent multiple training epochs with a batch size of 32. It achieved its highest accuracy at 500 epochs, registering an average geospatial RMSE of 0.0166 for the elephant Seri GPS observations dataset.

### 6.2. PoachNet Evaluation

To evaluate predictive methods on elephant Seri GPS collar data, we used its dataset in CSV format containing (date, time, longitude, latitude) features. The goal was to predict spatialtemporal coordinates (date, time, longitude, latitude) and compare the performance of three models: linear regression, polynomial regression, and Vector Autoregression (VAR). The performance was assessed using the average Root Mean Square Error (RMSE).

### 6.3. Data Preprocessing

The independent variables in this analysis are the input features used to make predictions. These include the day, month, and year, all of which are extracted from the ‘LocalDate’. These temporal features provide the contextual information necessary for the models to make accurate predictions.

The dependent variables, or targets, are the outputs the models aim to predict. These include geospatial coordinates such as latitude (‘lat’) and longitude (‘long’), as well as temporal features like the day, represented as a numeric value indicating the day of the month, and time, converted into a numeric representation of seconds since midnight (e.g., ‘12:34:56’ becomes ‘45,296’ s). Together, these outputs capture both spatial and temporal dynamics.

The data were divided into training and testing sets to ensure fair evaluation, and models were trained and tested sequentially to respect the temporal structure of the data.

### 6.4. Models and Results

1. Linear Regression: Applied simple linear regression using day as the predictor for both lat and long. Evaluated using 5-fold time-series cross-validation.
2. Polynomial Regression: Incorporated polynomial features (degree 4) to account for non-linear relationships. Similarly validated with 5-fold time-series cross-validation.
3. Vector Autoregression (VAR): Used both latitude and longitude as a multivariate time-series for temporal forecasting. Reserved a portion of the dataset for out-of-sample prediction and RMSE calculation.

The RMSE results for all models are summarized in Table 2. From the negatively-oriented RMSE scores (where a lower score indicates better performance), the linear regression model demonstrated strong performance for predicting latitude and longitude, with RMSE values of 0.123 and 0.164, respectively. Notably, the linear regression model also performed exceptionally well for predicting the day, achieving an RMSE close to zero, but struggled with time predictions, yielding an RMSE of approximately 25,186 s.

The polynomial regression model, while offering a more complex representation, exhibited higher RMSE values for latitude (2.396) and longitude (1.050) predictions. It achieved a near-perfect prediction for the day (RMSE: 7.2 × $10^{-6}$) but produced the highest error for time predictions, with an RMSE of 483,988 s. This suggests that the added complexity of the polynomial model may have led to overfitting or inefficiency for these particular features.

In comparison, the VAR model excelled in predicting longitude, with the lowest RMSE of 0.089. It also performed reasonably well for latitude predictions (RMSE: 0.222) but struggled with day and time predictions, with RMSE values of 8.69 and 25,093 s, respectively. These results indicate that the VAR model effectively captures temporal dependencies for geospatial coordinates but may require additional feature refinement for accurate temporal predictions.

**Figure 7:**
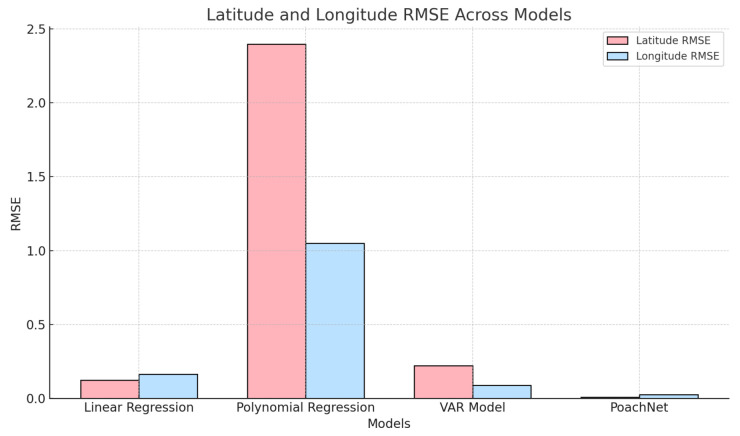
The chart visually compares the performance of PoachNet with other predictive models, specifically focusing on their Root Mean Square Error (RMSE) values, a standard measure of prediction accuracy. The other models include ‘Linear Regression’, ‘Polynomial Regression’, and ‘VAR’.

However, PoachNet (a neural network built with TensorFlow and Keras) trained on the same elephant Seri data but in an ontology-based knowledge graph, outperformed all other models. PoachNet achieved test RMSE values of 0.0247 for longitude, 0.0084 for latitude, 0.0123 for ‘localDate’, and 0.0086 for ‘localTime’. These results demonstrate the effectiveness of leveraging a knowledge graph representation and deep learning methods for highly accurate geospatial and temporal predictions. Figure 7 presents a comparison of the RMSE scores of PoachNet against those of other prediction models. Codes are available on Github https://github.com/Naeima/PoachNet accessed on 11 December 2024.

## 7. Discussion

The loss of forest elephants and their dispersal from poaching or habitat loss and fragmentation [2] could lead to reduced forest diversity, the inability of elephants to colonise new or deforested areas, and potentially reduced carbon stocks. Combating poaching in Sabah is a priority, and various organizations, including the Sabah Wildlife Department and Sabah Forestry Department with the support of Danau Girang Field Centre and WWF-Malaysia, are working to protect the Bornean elephant and many other species. The Bornean Elephant Action Plan for Sabah 2020–2029 is a ten-year plan approved by the state government of Sabah to conserve the Bornean elephant population and many other species. The plan has four main objectives: improve protection and reduce elephant deaths, improve landscape connectivity and permeability, ensure the best ex-situ practices for elephant management and conservation, and monitor and predict elephant population trends.

This research differentiates itself from existing views by integrating heterogeneous wildlife data with deep learning on an ontology-based knowledge graph. While prior approaches have primarily focused on specific aspects, such as social network analysis, multimedia data mining, or hierarchical models on ranger patrol data, this methodology offers an interconnected understanding of wildlife dynamics. The results highlight that while linear regression is well-suited for simple relationships in this dataset, and the VAR model shows promise for geospatial predictions, PoachNet surpasses them significantly, showcasing the potential of neural networks combined with knowledge graph techniques. Polynomial regression, despite its theoretical flexibility, did not outperform the simpler models and may require better feature engineering to improve its effectiveness.

PoachNet predictions can assist in the strategic resource allocation for anti-poaching efforts. It can also guide the decision to deploy ground truth sensors and motion-activated camera traps in areas most likely to have anticipated poaching crimes.

Research challenges include semantic heterogeneity among diverse data sources, which risks the consistent representation of information in the knowledge graph. Scalability issues may emerge as the knowledge graph expands, necessitating careful resource management. To address scalability issues in our knowledge graphs, several strategies can be recommended. Partitioning the graph into manageable subgraphs and using distributed triple-store databases like Stardog, Neo4j or Amazon Neptune can enhance processing efficiency. Incremental updates minimise reprocessing, while graph compression and summarisation reduce storage demands. Scalable cloud-based storage, optimized query processing with indexing, and the use of high-performance graph algorithms further improve performance. Edge computing can preprocess data near collection points, reducing bandwidth and latency.

Optimizing the deep learning algorithms in PoachNet to enhance predictive performance while minimising computational costs can be achieved through model compression techniques such as pruning and quantisation [51]. These approaches reduce the size of deep neural networks while maintaining accuracy, enabling faster inference, reduced storage requirements, and lower training costs. Techniques like low-rank decomposition, knowledge distillation, and lightweight model design can further streamline model deployment, making them more efficient for use in resource-constrained environments [52].

PoachNet can be expanded by integrating additional wildlife data sources such as acoustic sensors, satellite imagery, and crime intelligence. Acoustic sensors can detect gunshots, elephant vocalisations, or vehicle noises associated with poaching crimes. Satellite imagery can monitor changes in habitat, detect unauthorised human activity, and assess landscape connectivity. Crime intelligence data can add historical context, identifying patterns in poaching incidents and aiding in predicting future hotspots.

## 8. Conclusions

This study introduced PoachNet, a novel tool integrating Semantic Web technologies and deep learning to predict wildlife dynamics and poaching crime. By combining diverse wildlife data into an ontology-based knowledge graph enriched with rule-based reasoning, PoachNet provided a dynamic, hybrid predictive solution for conservation. Custom-built dataset and advanced neural network models accurately predicted elephant geo-locations and potential poaching incidents, achieving an average geospatial RMSE of 0.0166, surpassing state-of-the-art methods. This approach predicts future elephant geo-locations and uses this information to infer poaching risks based on proximity to identified hazardous areas. PoachNet equips biologists and conservationists with advanced tools for spatiotemporal poaching predictions, offering a transformative paradigm for wildlife crime prevention. While challenges such as semantic heterogeneity, data sensitivity, and ecosystem dynamics persist, the public release of the ontology-based knowledge graph and source code demonstrates our commitment to transparency and collaboration, encouraging the research community to collaborate with us and build upon this work.

## Figures and Tables

**Table 1:** Summary statistics of GPS data and related metrics.

	Lat	Long	Temperature	ExtTemp	Activity	Speed	Direction	Cov	HDOP	Distance	Count
count	9168.00	9168.00	9168.00	9168.0	9168.0	9168.0	9168.0	9168.00	9168.00	9168.00	9168.00
mean	5.20	118.66	29.20	0.0	0.0	0.0	0.0	1.23	2.21	273.89	6782.50
std	0.10	0.11	1.93	0.0	0.0	0.0	0.0	1.89	2.07	429.72	2646.72
min	5.01	118.44	−37.00	0.0	0.0	0.0	0.0	0.00	0.00	0.00	2199.00
max	5.38	118.95	60.50	0.0	0.0	0.0	0.0	5.00	21.00	8572.00	11,366.00

**Table 2:** Comparison of RMSE between PoachNet and state-of-the-art models.

Model	Latitude RMSE	Longitude RMSE	Average RMSE
Linear Regression	0.123	0.164	0.144
Polynomial Regression	2.396	1.050	1.723
VAR Model	0.222	0.089	0.156
PoachNet	0.0084	0.0247	0.0166

## Data Availability

The data are shared in github.com/Naeima/PoachNet.
